# Uptake and transport of B_12_-conjugated nanoparticles in airway epithelium^☆^

**DOI:** 10.1016/j.jconrel.2013.08.028

**Published:** 2013-11-28

**Authors:** Robyn Fowler, Driton Vllasaliu, Franco H. Falcone, Martin Garnett, Bryan Smith, Helen Horsley, Cameron Alexander, Snow Stolnik

**Affiliations:** a Division of Drug Delivery and Tissue Engineering, School of Pharmacy, University of Nottingham, Nottingham NG7 2RD, UK; b Division of Molecular and Cellular Science, School of Pharmacy, University of Nottingham, Nottingham NG7 2RD, UK; c UCB Pharma, 208 Bath Road, Slough, Berkshire SL1 3WE, UK

**Keywords:** Vitamin B_12_, Airway epithelium, Cubilin, Calu-3 cells, Epithelial nanoparticle transport

## Abstract

Non-invasive delivery of biotherapeutics, as an attractive alternative to injections, could potentially be achieved through the mucosal surfaces, utilizing nanoscale therapeutic carriers. However, nanoparticles do not readily cross the mucosal barriers, with the epithelium presenting a major barrier to their translocation. The transcytotic pathway of vitamin B_12_ has previously been shown to ‘ferry’ B_12_-decorated nanoparticles across intestinal epithelial (Caco-2) cells. However, such studies have not been reported for the airway epithelium. Furthermore, the presence in the airways of the cell machinery responsible for transepithelial trafficking of B_12_ is not widely reported. Using a combination of molecular biology and immunostaining techniques, our work demonstrates that the bronchial cell line, Calu-3, expresses the B_12_-intrinsic factor receptor, the transcobalamin II receptor and the transcobalamin II carrier protein. Importantly, the work showed that sub-200 nm model nanoparticles chemically conjugated to B_12_ were internalised and transported across the Calu-3 cell layers, with B_12_ conjugation not only enhancing cell uptake and transepithelial transport, but also influencing intracellular trafficking. Our work therefore demonstrates that the B_12_ endocytotic apparatus is not only present in this airway model, but also transports ligand-conjugated nanoparticles across polarised epithelial cells, indicating potential for B_12_-mediated delivery of nanoscale carriers of biotherapeutics across the airways.

## Graphical abstract

The vitamin B_12_ endocytic system is present in the airways, being able to transport B_12_-functionalized nanoparticles transepithelially. B_12_-nanoparticles display enhanced apical-to-basolateral transport, and a different trafficking behaviour to soluble B_12_.

**Figure:**
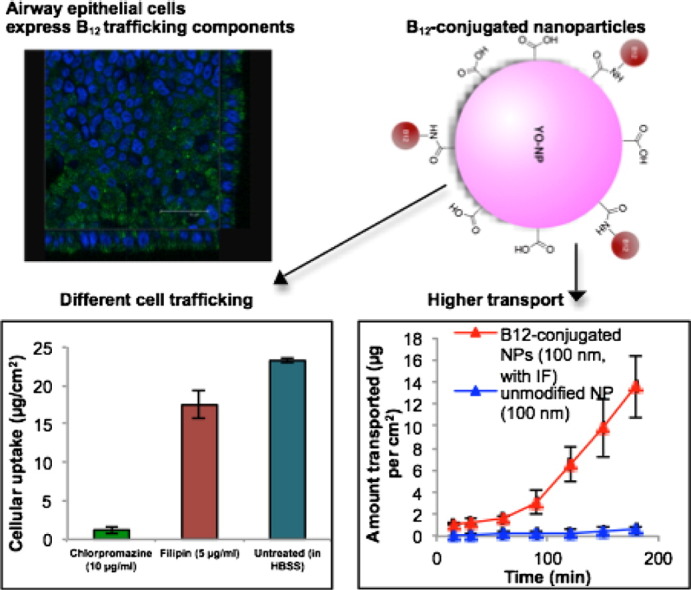

## 1 Introduction

Considering the recent emergence of biotherapeutics, developments in non-invasive delivery options for this class of medicines have been disappointing, with the parenteral method remaining the predominant administration route. Non-invasive administration *via* the mucosal surfaces (*e.g.* gut or airway epithelia) is highly desirable, but at the same time a formidable task, primarily due to the poor absorption of biomacromolecules across the mucosae. Mucosal absorption of biomolecules could potentially be improved by inducing reversible ‘opening’ of the epithelial tight junctions, creating a temporarily accessible corridor for the therapeutic to traverse the epithelial barrier [1–3]. This strategy however may be limited to relatively small biomolecules (peptides or small proteins), whilst systemic delivery of larger biotherapeutics (*e.g.* antibodies or PEGylated antibody fragments) through this approach is shown to be inefficient [4]. Furthermore, the longer-term implications of repeated opening of the tight junctions are currently unclear. Mucosal absorption of the therapeutic *via* transport through the epithelial cells (*i.e.* the transcellular route) would potentially overcome this problem.

A number of biological transport pathways operate at mucosal surfaces, ‘shuttling’ various molecules including nutrients (*e.g.* vitamin B_12_ [5]) and endogenous macromolecules (*e.g.* albumin and IgG [6]) across the epithelium. These physiological pathways could potentially be exploited for transmucosal delivery of biomolecules, delivered encapsulated within appropriately decorated nanoparticulate carriers [7,8].

The present work investigated whether the vitamin B_12_ (herein referred to ‘B_12_’) pathway could carry decorated nanoparticles across the airway mucosa. The B_12_ transcytotic pathway has demonstrated potential for intestinal delivery of peptides and proteins [7,9–12] and its presence has been confirmed in intestinal cell lines, Caco-2 [13,14] and HT29 [15,16]. Importantly, the B_12_ pathway has also been shown to transport nanoparticles across intestinal Caco-2 cells [11,17,18]. Whilst the possibility of targeting the B_12_ transcytosis pathway for delivery of biotherapeutics and nanocarriers has been demonstrated in the intestinal epithelium, information on the presence and its functioning in the pulmonary system is sparse [19] and knowledge on the drug delivery potential of this epithelial transport route in the airway epithelium is currently not available.

Here we demonstrate the expression and functioning of the B_12_ transcytotic system in differentiated, polarised culture of bronchial Calu-3 cells, and extend this to probing whether this pathway can be exploited for transairway delivery of nanoparticles as model therapeutic carriers. The objective of this work stems from other published studies by our group, which showed folate receptor expression and its endocytotic activity in airway epithelial cells [20,21]. Initial experiments assessed the expression of relevant pathway components: intrinsic factor (IF)-B_12_ receptor (‘cubilin’), the transcobalamin II receptor (‘TCII receptor’) and transcobalamin II carrier protein (‘TCII protein’), the latter being responsible for the delivery of B_12_ out of the basolateral membrane of epithelial cells *in vivo*. We further show cellular internalisation and transepithelial transport of B_12_-conjugated nanoparticles and unravel the endocytic mechanisms involved in the trafficking of B_12_-decorated nanoparticles.

## 2 Materials and methods

Solid 1,1′-carbonyldiimidazole (CDI), anhydrous dimethyl sulfoxide, cyanocobalamin and N-hydroxy-succinimide (NHS) were purchased from Acros® (Belgium). 1-Ethyl-3-[3-dimethylaminopropyl]carbodiimide (EDAC) was obtained from Calbiochem® (UK). Unless otherwise stated, all other chemicals and materials were supplied by Sigma-Aldrich® (UK) or Fisher Scientific® (UK).

### 2.1 Preparation of the α-ω-aminohexylcarbamate derivative of cyanocobalamin

The α-ω-aminohexylcarbamate derivative of cyanocobalamin was prepared using a method described previously [18]. Briefly, solid CDI (260 mg, 0.32 mmol) was added to cyanocobalamin (1.0 g, 0.148 mmol) previously dissolved in anhydrous dimethyl sulfoxide. The mixture was stirred for up to 2 h at 30 °C, followed by the addition of dry 1,6-hexanediamine (314 mg, 0.54 mmol) and stirring of the mixture at room temperature over 24 h. The mixture was poured into ethyl acetate (30 ml) and left to stand. Following centrifugation and decanting of the supernatant, the residue was sonicated for 5 min in acetone (50 ml). The resulting precipitate was filtered and the solid washed in acetone. The crude product was purified by silica column chromatography (45% v/v 2-propanol, 30% v/v n-butanol, 2% v/v ammonia and 25% v/v water) followed by lyophilisation.

### 2.2 Preparation of B_12_-conjugated nanoparticles

1 ml of fluorescent carboxylate Yellow Orange (YO, fluorescence spectra equivalent to rhodamine) polystyrene nanoparticles of 50 nm, and 100 nm (2.69 and 2.6% w/v aqueous suspension, respectively; PolySciences Inc., Germany) were modified with the α-ω-aminohexylcarbamate B_12_ derivative (10 mg) by activation with 1-ethyl-3-[3-dimethylaminopropyl]carbodiimide (EDAC), in the presence of N-hydroxy-succinimide (NHS). The reaction was allowed to proceed for 5 h followed by incubation with glycine (100 mg/ml) in 50 mM carbonate buffer, pH 9.5, to block residual activated carboxyl sites. The particles were then dialyzed extensively against distilled water over 24 h, whilst exchanging the water at regular intervals.

### 2.3 Characterisation of B_12_-conjugated nanoparticles

Extensive characterization of the B_12_ derivative and the conjugated nanoparticles (*e.g.* size and surface charge determination pre- and post-B_12_ conjugation) was described in a recent publication by our group [18].

### 2.4 Cell culture

Calu-3 cells (American Type Culture Collection, USA) were cultured using Eagle's Minimum Essential Medium (LGC standards, UK), supplemented with penicillin (100 units/ml), streptomycin (0.1 mg/ml), amphotericin (0.25 μg/ml) and Foetal Bovine Serum (FBS, 10% v/v). Cells were seeded on Transwell® inserts (12 mm diameter, 0.4 μm pore size; Corning Life Sciences, Holland) at 10^5^ cells/cm^2^ and cultured for 14–21 days, with medium replacement every other day. Calu-3 cells were cultured using air-interfaced culture (AIC) conditions (known to promote mucus secretion), which were created on day 2 post-seeding on filter supports. Cell confluence and cell layer integrity following nanoparticle exposure were confirmed by transepithelial electrical resistance measurements (TEER) using an EVOM (World Precision Elements, USA) voltohmmeter. TEER post-nanoparticle incubation did not drop significantly (Supporting information, Fig. S1), suggesting no effect on the integrity of the cell layers.

### 2.5 mRNA expression of cubilin and TCII receptor in Calu-3 cells

#### 2.5.1 mRNA isolation and cDNA synthesis

The μMACS™ (Miltenyi Biotec, UK) was used to extract mRNA and synthesise cDNA from Calu-3 cells and Caco-2 cells (used as a control) cultured on Transwell® supports for 21 days. This single-step technique that captures mRNA magnetically with oligo-dT magnetic beads was conducted according to the manufacturer's recommended protocol. Note, that 50 μl of sscDNA was collected per tube of which 20 μl was collected and pooled for primer optimisation.

#### 2.5.2 Primer design

The ensemble database was consulted for primer design (www.ensembl.org). PCR primers for human cubilin (HsCUBN_FOR): 5′-TCCGGCAGACATTGGGGCCT-3′ and (HsCUBN_REV): 5′-TCCGTGACCCTGCGGTGAGT-3′ were designed from human cubilin sequence, National Center for Biotechnology Information (NCBI) Reference Sequence NM_001081.3. PCR primers for human TCII receptor (CD320) (HsCD320_FOR): 5′-CACCCACCAAGTTCCAGTGCCG-3′ and (HsCD320_REV): 5′-GTTCCACAGCCGAGCTCGTCG-3′ were designed from human CD320 sequence, NCBI Reference Sequence NM_016579.3. Primers for cubilin span an exon–exon junction with an intron size of 18,192 basepairs (forward primer) and 3947 basepairs (reverse primer). The primers for CD320 include an intron with a size of 942 bp. Both primer pairs were blasted against the human genome (Primer-Blast on NCBI Blast) and identified one single amplicon of the correct size in each case.

#### 2.5.3 Polymerase chain reaction (PCR)

Standard PCR ready-to-use Master Mix (REDTaq® ReadyMix™ PCR Reaction Mix, Sigma-Aldrich, UK) was used to prepare the PCR reaction mixture. The final reaction mixture consisted of 10 μl PCR Master-Mix, 1 μl each of forward and reverse primers for the gene of interest (5 μM each final concentration), 1 μl DNA template (extracted cDNA of filter-cultured Calu-3 and Caco-2 cells) and 7 μl nuclease free water (Roche, UK), making a final volume of 20 μl per microtube. PCR amplification of cDNA for human cubilin was performed using the following sequential cycles: initial heating at 94 °C for 5 min, 94 °C for 30 s, annealing at 60 °C for 45 s, extension at 72 °C for 90 s, and this cycling repeated 34 times, followed by a final extension at 72 °C for 5 min to ensure completion of all strands. A similar PCR programme was followed for human TCII receptor but with denaturation steps at 58 °C. Reactions were carried out in a PTC-200 Peltier Thermal Cycler (MJ Research, UK). The reaction mixes were incubated at 4 °C prior to DNA agarose gel electrophoresis.

#### 2.5.4 DNA agarose gel electrophoresis

The PCR products of 602 bp and 343 bp for cubilin and TCII receptor, respectively, were analysed by DNA Agarose gel electrophoresis. Agarose gels were prepared using a well-established protocol, developed by the group and adapted from Sambrook and Russell [22]. Briefly, 1% (w/v) agarose gels were prepared using analytical grade agarose (Promega, UK) in 0.5X TBE buffer (1 M Tris–HCl; 1 M Boric Acid; 0.05 M EDTA; pH 8.0) with the addition of ethidium bromide (10 μg/ml). 8 μl (0.8 μg) of TriDye 100 bp DNA Ladder was loaded per gel lane (2 lanes per gel on either side of DNA samples). The DNA samples were loaded (15–20 μl per gel lane) and the gels were run in 0.5X Tris/Borate/EDTA (TBE) buffer and electrophoresis was performed using a horizontal gel apparatus (Fisher Scientific, UK) at 100–110 V. DNA fragments were visualised on a UV transilluminator with Gene Genius Bio imaging software (UVP, USA).

### 2.6 Immunostaining for cubilin, transcobalamin II receptor and transcobalamin II protein

Following culture as polarised layers, Calu-3 cells were fixed using 4% w/v paraformaldehyde (in PBS) for 10 min at room temperature. Cells were then washed with PBS, followed by incubation with 1% w/v BSA in PBS for 1 h. The cells were then subjected to 30 min incubation with rabbit, anti-human cubilin H-300 (Santa Cruz Biotechnology, Inc.), rabbit, anti-human CD320 (TCII receptor, Abcam) or rabbit transcobalamin II H-260 (Santa Cruz Biotechnology, Inc.), all diluted 1:50 with 1% w/v BSA/PBS. The cells were then washed extensively with PBS and incubated with the secondary antibodies (chicken anti-rabbit Alexa-Fluor 488 for TCII receptor, and goat, anti-rabbit IgG-rhodamine, for cubilin and TCII), diluted 1:100 (in 1% w/v BSA/PBS) for another 30 min. In the control experiment cell layers were incubated with the secondary antibodies only. Following a final wash step, cell nuclei were stained with Hoechst 33342 (0.1 mg/ml), the cells washed and the filter membrane excised. Cells were mounted on a slide using 1,4-diazabicyclo[2.2.2]octane (DABCO) (1% diluted in 9:1 glycerol:PBS) and covered with a glass cover slip. Confocal imaging was carried out using a Leica SP2 CLSM-Micro4 confocal microscope (Leica, Germany).

### 2.7 Cellular uptake and transport studies of B_12_-conjugated nanoparticles

Culture medium was removed from filter-cultured Calu-3 cells (cultured using AIC conditions) and replaced with Hank's Balanced Salt Solution (HBSS), buffered with 2-[4-(2-hydroxyethyl)piperazin-1-yl]ethanesulfonic acid (HEPES, 20 mM); cell layers were incubated with HBSS for 45 min. Unmodified YO nanoparticles (50, 100 nm) and B_12_-conjugated nanoparticles were suspended in HBSS/HEPES to achieve a final concentration of 400 μg/ml. Approximately 0.115 μg of recombinant human intrinsic factor (rHUIF, Autogen Bioclear Ltd) was added per 1 ng of B_12_ and the solution was incubated at 37 °C prior to application to cells. Following a routine measurement of TEER (to assess cell layer integrity and hence their suitability for inclusion in the experiments) nanoparticle suspensions (0.5 ml) were applied to the apical chamber of quadruplicate wells and the Calu-3 cultures incubated at 37 °C over 3 h. B_12_-conjugated nanoparticles were also applied to the cells in the absence of IF. At 30 minute sampling intervals, 100 μl was removed from the basolateral side and analysed for fluorescence. Sample losses were replaced with 100 μl of HEPES/HBSS to maintain the basolateral volume constant. Internalised fluorescence (indicating nanoparticle uptake) was determined by cell lysis using 0.2% Triton X-100 (Fluka) (10 min incubation). Cell uptake of nanoparticles was quantified by fluorescence (Dynex, microplate reader, 529 nm/546 nm) using calibration curves (conducted in triplicates, in the medium replicating the experimental conditions). Soluble B_12_ transport studies were conducted in a similar manner to nanoparticle transport experiments, with B_12_ quantified by UV (350 nm, Beckman Coulter DU 800 UV spectrophotometer).

### 2.8 Immunostaining for clathrin and caveolin-1

For immunostaining experiments, culture medium was removed and cells incubated with HBSS for 45 min. The cell monolayers were then fixed with 4% paraformaldehyde (diluted in PBS) for 10 min at room temperature. For these studies, an additional permeabilization step was conducted *via* the addition of 0.2% Triton X-100 for 10 min. The cells were washed with PBS, before incubation with 1% BSA/PBS (for 1 h). The primary antibody for caveolin-1 (rabbit, anti-human caveolin-1 H-97 IgG; Santa Cruz Biotechnology, Inc., USA) was diluted 1:50 with 1% BSA/PBS and incubated with the cells for 30 min. For clathrin immunostaining, rabbit, anti-human clathrin IgG (Abcam®, UK) was diluted 1:200 in 1% BSA/PBS. The cells were washed 3 times with PBS and incubated with the secondary antibody (goat, anti-rabbit IgG-rhodamine and goat, anti-rabbit IgG-FITC for caveolin-1 and clathrin, respectively) diluted 1:100 (in 1% BSA/PBS) for another 30 min. The control cell layers were incubated with the appropriate secondary antibody only. Following a final wash step, the cell nuclei were stained with Hoechst 33342 (0.1 mg/ml), the cells washed and the filter membrane excised from the insert. Cells were mounted on a slide using 1,4-diazabicyclo[2.2.2]octane (DABCO) (1% diluted in 9:1 glycerol:PBS) and covered with a glass cover slip. Confocal imaging was carried out using a Leica SP2 CLSM-Micro4 confocal microscope.

### 2.9 Clathrin and caveolae inhibition studies

Filipin (5 μg/ml) and chlorpromazine (10 μg/ml) were used as inhibitors of specific endocytic pathways (caveolae and clathrin, respectively). Cells were treated with the inhibitors, in HBSS/HEPES, for 1 h at 37 °C prior to the addition of particles [23]. Unmodified and B_12_-conjugated nanoparticles (as IF composites and without IF) were applied in HBSS/HEPES (400 μg/ml) containing one of the above inhibitors and incubated for 3 h. Control experiments with known ligands for the clathrin- and caveolae- mediated pathways (FITC-transferrin at 100 μg/ml and cholera toxin-B-subunit at 5 μg/ml, respectively) were carried out as described previously [18]. To assess the trafficking route of soluble vitamin B_12_ and compare with that of B_12_-bearing nanoparticles, vitamin B_12_ (1 mg/ml), in the presence of IF, was also applied in conjunction with the same inhibitors and cell uptake and transport determined *via* UV-absorbance at 350 nm.

### 2.10 Statistical analysis

All experiments were carried out using triplicate samples and were repeated three times. One way analysis of variance (ANOVA) followed by Bonferroni post-hoc test was applied for comparison of group means of three or more groups, whilst Student's *t*-test was used for comparison of two groups. Data shown as the mean ± SD of three different experiments. p values of < 0.05 were considered statistically significant. *, ** and *** denote p < 0.05, p < 0.01 and p < 0.001, respectively, whilst ‘ns’ indicates non-significant.

## 3 Results

### 3.1 Cubilin and TCII receptor expression in Calu-3 cells

#### 3.1.1 mRNA expression

**Fig. 1:**
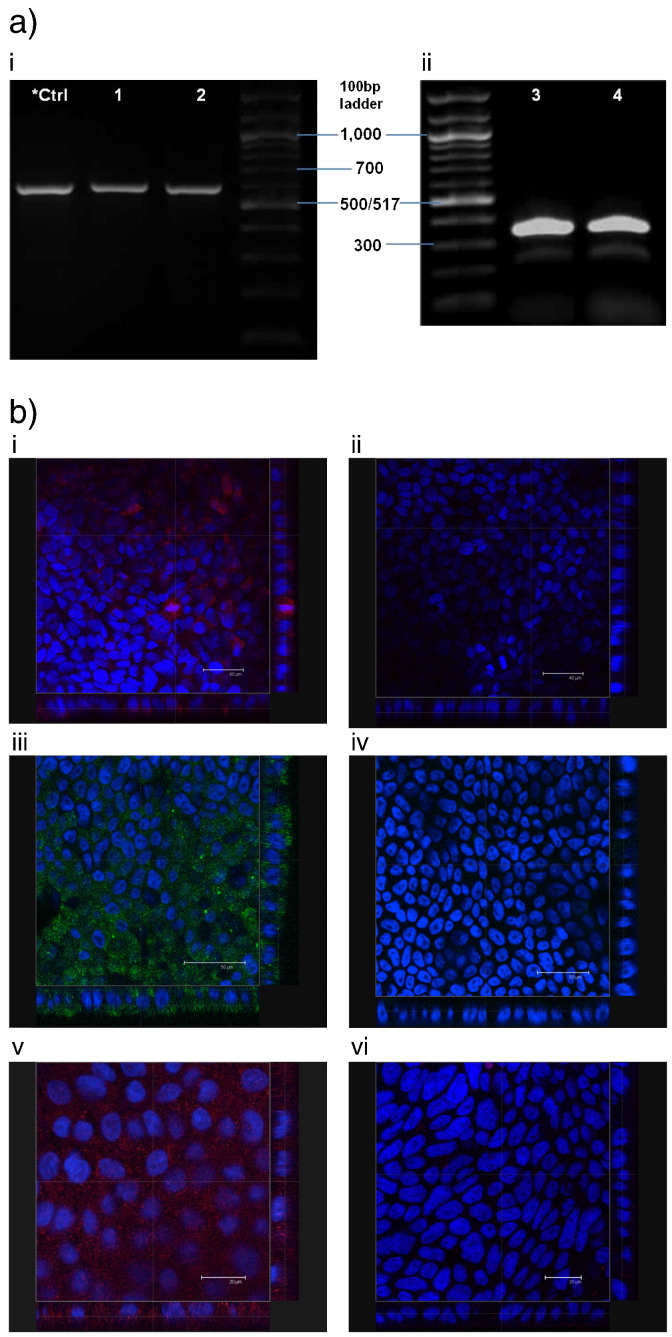
Cubilin, TCII receptor and TCII protein expression in polarised Calu-3 cells on day 21 of culture. a) mRNA expression of (i) cubilin (lanes 1 & 2) and (ii) TCII receptor (CD320) (lanes 3 & 4). *Ctrl denotes PCR product obtained from Caco-2 cell monolayer. b) Immunostaining for cubilin (i–ii), TCII receptor (iii–iv) and TCII (v–vi). i) Red signal from Rhodamine-labelled goat, anti-rabbit IgG antibody to rabbit anti-human cubilin H-300 primary antibody. ii) Control experiment where cells were treated with Rhodamine-labelled goat, anti-rabbit IgG only (*i.e.* treatment with the primary antibody was omitted). iii) Cells immunostained for TCII receptor with rabbit, anti-human CD320 antibody and chicken, anti-rabbit Alexa Fluor 488 secondary antibody. iv) Control experiment showing cells treated with chicken, anti-rabbit Alexa Fluor 488 secondary antibody only. v) Cells immunostained for TCII with rabbit, anti-human TCII H-260 and Rhodamine-labelled goat, anti-rabbit IgG secondary antibody. vi) Control experiment in which cells were treated with Rhodamine-labelled goat, anti-rabbit IgG only. Blue = cell nuclei stained with Hoechst. Micrographs shown as overlay images showing both blue and red or blue and green channels.

PCR and gel electrophoresis analysis of differentiated Calu-3 cells (cultured on permeable supports as polarised layers), conducted to determine mRNA expression of cubilin (human CUBN gene) and the gene encoding TCII receptor (CD320), is shown in Fig. 1a. The data confirm the expression of both cubilin (i) and TCII receptor (ii) (cDNA amplification products of 602 and 343 bp, respectively). A secondary fragment (~ 260 bp) with weak intensity, in addition to the band detected at 343 bp (for CD320), was also detected, which possibly results from an alternatively spliced transcript variant encoding a different isoform.

The expression of cubilin, TCII receptor and TCII protein, at protein level, is demonstrated by immunostaining. This is shown in Fig. 1b. Considering the expression of cubilin, fluorescence signal was detected and appears distributed across the depth of the cell layer in the sample treated with both primary and secondary antibodies (Fig. 1b i), implying a non-polar receptor distribution. Fluorescence was not apparent in the control experiment where treatment with the primary antibody was omitted (Fig. 1b ii). Immunostaining for TCII receptor reveals intense staining, indicating its expression in Calu-3 cells (Fig. 1b iii). No fluorescence was detected in the control experiment (*i.e.* treatment with the secondary antibody only, Fig. 1b iv). Immunostaining for TCII protein, a carrier protein involved in the binding and transport of B_12_ out of the enterocytes *in vivo*, indicates its presence in Calu-3 cells (Fig. 1b v), whilst fluorescence signal was largely absent in the relevant control experiment (Fig. 1b vi).

### 3.2 Characterisation of B_12_-conjugated nanoparticles

The characterisation of amino-derivatized B12 by means of nuclear magnetic resonance (NMR), mass spectrometry (MS), and high-performance liquid chromatography (HPLC), as well as characterisation of conjugated nanoparticles is shown in our recently published work, which employed the same B_12_-modified nanoparticles [18]. Briefly, nanoparticle size and surface charge before and after B_12_ conjugation were determined by dynamic light scattering and zeta potential measurements, demonstrating a change in both parameters post-B_12_ conjugation. The presence of B_12_ on the surface of conjugated polystyrene nanoparticles was confirmed by fluorescence quenching, based on the fluorescence quenching ability of B_12_, which was previously demonstrated in work by our group [18] and others [24,25]. This is shown by experiments determining the fluorescence of physical mixtures of nanoparticles and B_12,_ as well as B_12_-conjugated nanoparticles before and after dialysis (performed to separate soluble B_12_ from unconjugated nanoparticles). The data shown in Supporting information, Fig. S2, demonstrates that B_12_-conjugated nanoparticles displayed a lower fluorescence intensity compared to unmodified nanoparticles and that fluorescence remained unchanged following dialysis (unlike physical mixtures of soluble B_12_ and nanoparticles).

### 3.3 Cell uptake and transport of B_12_-conjugated nanoparticles

**Fig. 2:**
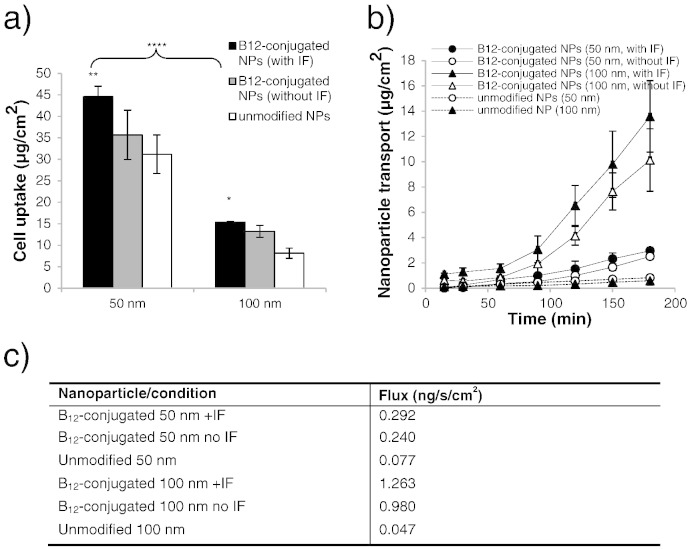
Cell uptake and transport of B_12_-conjugated nanoparticles in Calu-3 layers: a) Cell uptake of B_12_-conjugated nanoparticles (50 nm and 100 nm) in the presence and absence of intrinsic factor (IF) and unmodified nanoparticles. b) Transport of B_12_-conjugated nanoparticles (50 and 100 nm) in the presence and absence of IF, and unmodified nanoparticles. c) Nanoparticle transport flux. Data represents the mean ± SD.

Fig. 2a provides a comparison of cell uptake of B_12_-conjugated nanoparticles with unmodified counterparts of the same pre-conjugation diameter. Following a three-hour incubation period, the uptake of B_12_-conjugated nanoparticles by Calu-3 cells was larger compared to unmodified nanoparticles. This trend applied to both 50 nm (45 μg *versus* 31 μg for B_12_-conjugated and unmodified nanoparticles, respectively) and 100 nm systems (15 μg *versus* 8 μg). Testing the effect of intrinsic factor (IF) on the cell uptake of B_12_-conjugated nanoparticles, Fig. 2a also shows that the presence of IF in the system modestly promoted the uptake of B_12_-nanoparticles, with the effect being marginally larger for 50 nm nanoparticles (1.25-fold increase), compared to 100 nm nanoparticles (1.16-fold).

Mirroring the cell uptake data, the results showing nanoparticle transport across polarised cells (Fig. 2b and c) revealed that B_12_-conjugated nanoparticles traversed Calu-3 cell layers more efficiently than the unmodified counterparts, regardless of their size. The difference in transport between B_12_-bearing and unmodified nanoparticles of the same nominal diameter was notable: 0.240–0.292 ng/s/cm^2^ *versus* 0.077 ng/s/cm^2^ (up to 3.8-fold difference) and 0.98–1.263 ng/s/cm^2^ *versus* 0.047 ng/s/cm^2^ (up to 27-fold difference) for 50 nm and 100 nm nanoparticles, respectively. Transport of nanoparticles over 3 h was somewhat lower in the absence of IF and the level of transport was significantly larger for 100 nm, relative to 50 nm nanoparticles, with or without the presence of IF in the system. Cumulative apical-to-basolateral transport of 100 nm nanoparticles at the end of the three-hour exposure reached high levels of approximately 15 μg — a similar level to the amount nanoparticles found intracellularly at this time point.

### 3.4 Cell uptake of B_12_-conjugated nanoparticles: confocal microscopy

Confocal micrographs of polarised Calu-3 cells following incubation with unmodified and B_12_-conjugated nanoparticles are presented in Supporting information, Fig. S3a and b, respectively. In both conditions the micrographs clearly show the presence of nanoparticle-emitting red fluorescence signal within the cells. Imaging in sections across the depth of the cells shows the distribution of fluorescence across the vertical plane of the cell layers (depicted on the right and bottom sides of the images), indicating nanoparticle presence deep within the cells, rather than simple association with the cell membranes. It must be noted that nanoparticle fluorescence signal was more apparent for unmodified nanoparticles compared to B_12_-conjugated nanoparticles (imaged under the same confocal microscopy settings), though quantitation of nanoparticle uptake demonstrated that the latter were taken up by the cells more efficiently (Supporting information, Fig. S3a). This is probably due to the prominent fluorescence quenching capacity of B_12_, as shown in Supporting information, Fig. S2.

### 3.5 Immunostaining for clathrin and caveolin-1

**Fig. 3:**
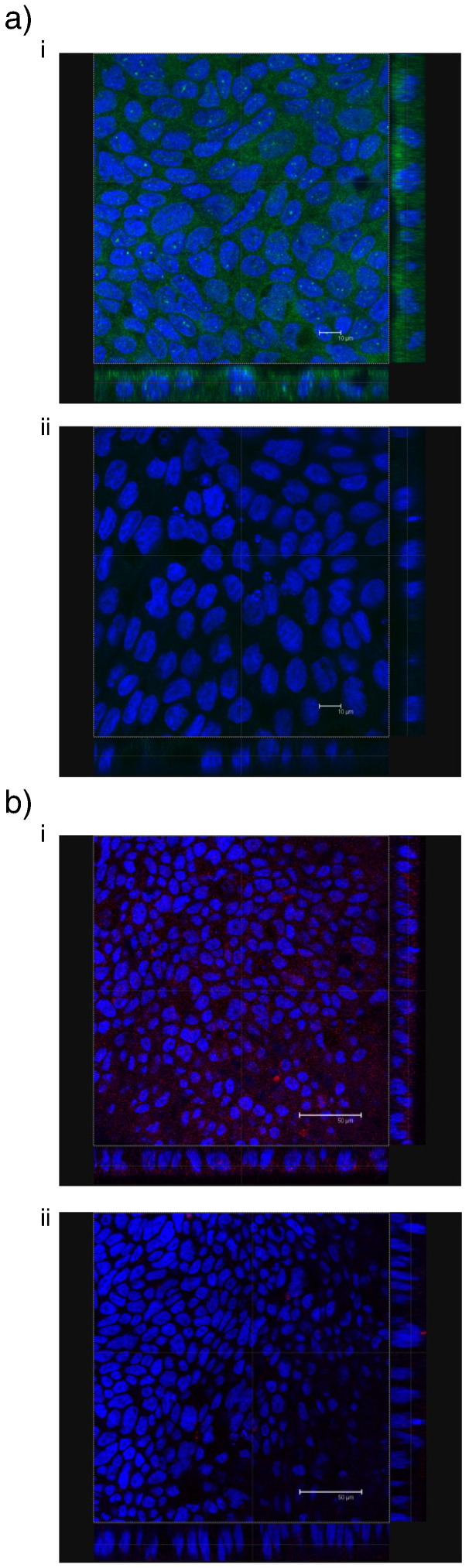
Expression of select endocytic components by immunohistochemistry in Calu-3 cells (as polarised layers). a) Expression of clathrin, as demonstrated by cell incubation with rabbit anti-clathrin primary antibody and goat, anti-rabbit IgG-FITC (i), and control monolayer treated with the secondary, goat, anti-rabbit IgG-FITC only (ii). b) Expression of caveolin-1, shown by treating cells with anti-human caveolin 1H-97, followed by goat, anti-rabbit IgG-Rhodamine (i), and control monolayer, incubated with goat, anti-rabbit IgG-Rhodamine only (ii). Cell nuclei were labelled with Hoechst 33342 (blue) in all cases. Immunostaining for all components was performed on day 21 of Transwell culture.

Calu-3 expression of key proteins relevant in two specific endocytic pathways, namely clathrin (implicated in the trafficking of the soluble B_12_-IF complex) and caveolae pathways, was confirmed by immunostaining. The data shown in Fig. 3 confirmed the expression of these proteins (clathrin and caveolin-1 in Fig. 3a and b, respectively) in the airway model, as shown by the presence of fluorescence signal in cell samples treated with the relevant antibodies and lack of fluorescence in control samples where exposure to the secondary, fluorescently-tagged antibody was omitted. This is in agreement with previously reported findings [26].

### 3.6 Epithelial trafficking of soluble B_12_

Studies assessing the uptake and transport route of soluble B_12_ in airway epithelial cell layers were undertaken using endocytic pathway-specific inhibitors, chlorpromazine and filipin. Specific inhibition of cell uptake pathways by these agents in Calu-3 cells was examined by assessing their effect on the uptake and transport of clathrin and caveolae pathway-selective ligands, transferrin [27,28] and cholera toxin B-subunit [29,30], respectively (Supplementary information, Fig. S4). Cell uptake of FITC-transferrin in the presence of chlorpromazine was inhibited by approximately 2-fold (Fig. S4a), whilst filipin induced a dramatic 21-fold decrease in the cellular internalisation of cholera toxin B-subunit (Fig. S4b).

**Fig. 4:**
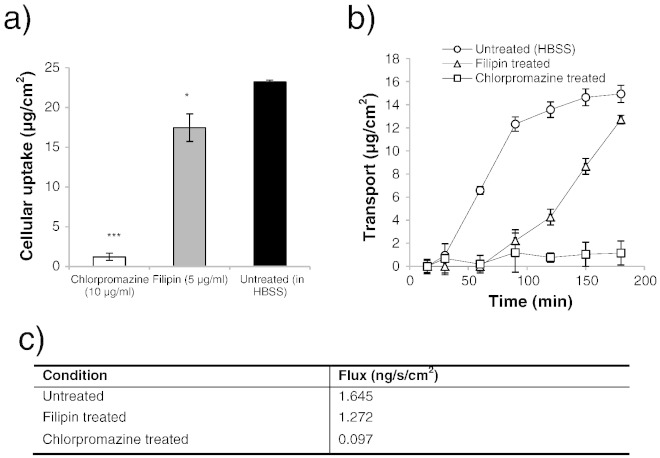
Effect of endocytic pathway-specific inhibitors on cell uptake and transport of soluble vitamin B_12_ in Calu-3 cell layers. a) Effect of chlorpromazine and filipin on cell uptake of B_12_. b) Effect of chlorpromazine and filipin on transport of B_12_ across Calu-3 layers. c) Soluble vitamin B_12_ transport flux. B_12_ was applied in combination with IF and quantified by UVAbs (350 nm). Data represents the mean ± SD.

Considering the effect of endocytosis inhibitors on cell uptake and transepithelial transport of soluble B_12_, Fig. 4 shows that both agents had an impact on cell uptake (Fig. 4a), with filipin causing a modest reduction in cell uptake (by approximately 25%) and chlorpromazine having a markedly more prominent effect with a 95% suppression of soluble B_12_ uptake. A similar observation was noted in transport studies: soluble B_12_ transport across filipin-treated cells was reduced compared to untreated cells, with a notable inhibitory effect in the initial phases of the experiment (Fig. 4b), which latter recovered. Overall transport inhibition by filipin, as determined from flux values (Fig. 4c), amounted to 23%. Chlorpromazine on the other hand induced a high level of transport inhibition (with a reduction in flux by 94%).

### 3.7 Airway epithelial cell trafficking of B_12_ conjugated nanoparticles

**Fig. 5:**
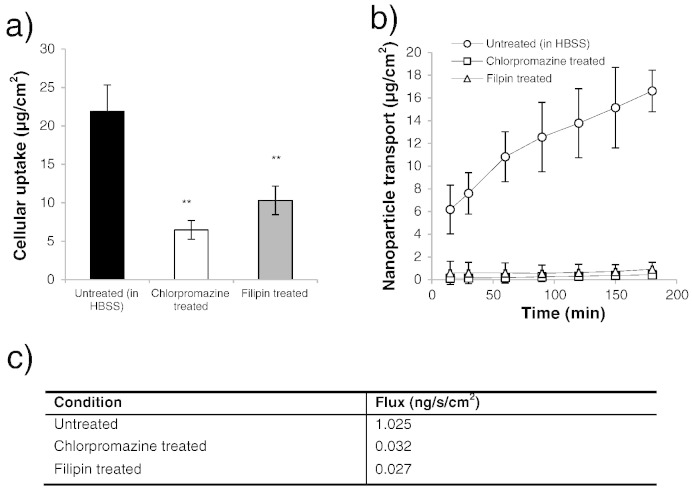
Effect of endocytic pathway-specific inhibitors on cell uptake and transport of B_12_-conjugated nanoparticles in Calu-3 cell layers. a) Effect of chlorpromazine and filipin on the cell uptake of B_12_-conjugated nanoparticles in Calu-3 layers. b) Effect of chlorpromazine and filipin on transport of B_12_-conjugated nanoparticles across Calu-3 layers. c) Nanoparticle transport flux. Cell uptake and transport studies were conducted with B_12_-conjugated nanoparticles of 100 nm diameter and in the presence of IF. Data represents the mean ± SD.

Cell uptake of 100 nm B_12_-nanoparticles was affected by both chlorpromazine and filipin treatment, producing a statistically significant reduction in the extent of nanoparticle uptake (Fig. 5a). Treatment with chlorpromazine induced a 3.4-fold decrease in nanoparticle internalisation (p = 0.002), whereas application of filipin caused a 2.1-fold reduction in the extent of uptake (p = 0.004). Transport data also illustrates the same phenomenon: after 3 h, cumulative amounts of B_12_-nanoparticles on the basal side in untreated cells reached ~ 17 μg, *versus* 0.9 and 0.4 μg, in filipin- and chlorpromazine-treated cells, respectively (Fig. 5b). Nanoparticle flux across the airway epithelium was reduced dramatically by 96.9% and 97.4% due to chlorpromazine and filipin treatment, respectively (Fig. 5c). These trends therefore suggest that cell uptake and transport of B_12_-conjugated nanoparticles was noticeably affected by the inhibition of either clathrin or caveolae-mediated routes in airway Calu-3 cells.

## 4 Discussion

Non-invasive delivery of biotherapeutics for a systemic effect is potentially feasible with administration *via* the mucosal surfaces. However, the epithelium presents an almost impenetrable barrier to the movement of macromolecular therapeutics into the systemic circulation. Natural epithelial transport pathways involved in the absorption of nutrients (*e.g.* vitamin B_12_ and folate) or regulation of immune responses (IgG/FcRn) have therefore been considered as potentially useful targets to ‘hijack’ for delivery of therapeutics and/or therapeutic carriers such as nanoparticles. However, not many known pathways are able to efficiently transport material transepithelially (*i.e.* transfer cargo across the cells, as opposed to merely internalising). To this end, the vitamin B_12_ absorption pathway has been relatively well investigated for its potential to ‘carry’ therapeutic biomolecules, as well as nanoparticles as drug carriers, transepithelially. However, these studies have been mainly conducted in Caco-2 cells, as an *in vitro* model of the intestinal epithelium, which is an obvious selection based on physiological absorption of dietary B_12_.

Information on the presence of the transcytotic machinery involved in transepithelial trafficking of B_12_ in the bronchial epithelium is, to our knowledge, not available, although the expression of cubilin in alveolar A549 cell line has recently been reported [19]. Therefore, a combination of molecular biology and immunostaining techniques was used to determine the presence of the relevant components at mRNA and protein level in airway Calu-3 cells. mRNA expression of cubilin and TCII receptor was analysed using PCR and gel electrophoresis. The data demonstrated the expression of both components (Fig. 1) in Calu-3 cells, as in Caco-2 cells, which were used as a control as the expression of cubilin and TCII receptor in these cells is widely acknowledged [31–33]. The expression of cubilin, TCII receptor and TCII protein in Calu-3 cells was also confirmed at protein level using immunofluorescence (Fig. 1b). The expression of these constituents of the B_12_ transport system at gene and protein level therefore indicates that vitamin B_12_ cell trafficking activity may operate in the airway epithelium.

Cubilin has been previously shown to be expressed in the renal proximal tubule and the visceral yolk sac [34], the placental cytotrophoblast and possibly other tissues such as thymus [35], in addition to the small intestinal epithelium. Whilst its expression in the renal proximal tubule, which exhibits a very extensive apical endocytic apparatus involved in the re-absorption and conservation of important molecules (such as vitamin B_12_) filtered in the glomeruli, is perhaps unsurprising, cubilin functionality in the airway epithelial cells is unclear. However, it presumably plays a physiological role in the uptake (rather than transepithelial transport) of B_12_ as an essential agent in key metabolic processes, similarly to the expression of folate receptor (another vitamin B subtype) in airway epithelial cells [21].

Following the demonstration that Calu-3 cells express vitamin B_12_ receptor, TCII receptor and TCII protein, we next determined the presence of other structural cell components participating in the process of endocytosis or transcytosis. This was deemed necessary as whilst the expression of cubilin and TCII receptor is required for the ligand–receptor binding stage of endocytosis, other components of the cell machinery are responsible for subsequent trafficking of B_12_. Further work therefore determined whether Calu-3 cells express relevant key proteins involved in two specific endocytic pathways, including the essential components of clathrin-coated pits, implicated in the internalisation of the soluble B_12_-IF complex, as well as caveolae (caveolin-1) as another route for material internalisation. The work confirmed the expression of these proteins by airway Calu-3 cells (Fig. 3), which was also shown in a previous study [26].

Amino derivatization of B_12_ and characterization of B_12_-bearing nanoparticles in terms of surface modification, size and charge, were reported in our recent work [18]. These nanoparticles, used as model therapeutic nanocarriers or nanomedicines, demonstrated interesting cell uptake and transport behaviours. B_12_-conjugated nanoparticles of 50 nm and 100 nm (nominal diameter) demonstrated improved cell uptake and transport across the cell layers, as compared to unmodified nanoparticles, with IF modestly influencing the extent of cell uptake (Fig. 2). Fluorescently-labelled B_12_-decorated nanoparticles were also clearly observed within Calu-3 cells following imaging by confocal microscopy (Supporting information, Fig. S3). In addition to demonstrating higher cell uptake, B_12_-conjugated nanoparticles transported across the cell layers notably more efficiently than unmodified nanoparticles, irrespective of their size. In the absence of IF, nanoparticle transport was somewhat reduced, indicating that IF plays a role (albeit minor) in the trafficking of B_12_-bearing nanoparticles. It is interesting to note that the transport of 100 nm nanoparticles was significantly larger relative to 50 nm nanoparticle, despite 50 nm B_12_-conjugated nanoparticles being internalised by the cells to a greater extent.

*In vivo*, native soluble B_12_ binds to IF and the B_12_-IF complex in turn binds to the cubilin receptor situated in the apical brush border membrane of the ileal mucosa [36]. Association of cubilin with megalin, a member of the LDL receptor-related family of endocytic receptors, [37,38] leads to internalisation of the entire cubilin-IF-B_12_ complex *via* clathrin-coated vesicles [39]. Our recent work showed that in intestinal Caco-2 cells soluble vitamin B_12_ internalises *via* clathrin-mediated endocytosis [18]. The mechanism of cell entry of soluble B_12_ in airway Calu-3 cells was also probed with chlorpromazine and filipin as cell trafficking inhibitors of clathrin- and caveolae-mediated endocytosis, respectively [23,29,30]. Soluble B_12_ internalisation and transport were significantly suppressed by the action of chlorpromazine. The caveolae inhibitor, filipin, displayed a modest effect on these phenomena (Fig. 4), with a 25% reduction in uptake and some influence in the pattern of transport over the three-hour experiment. The data therefore suggests that vitamin B_12_ is trafficked predominantly *via* a clathrin-mediated route in airway Calu-3 cells, which is in accord with previous reports related to other epithelial tissue [40,41], as well as our observations with intestinal Caco-2 cells [18]. However, whilst in intestinal Caco-2 cells caveolae played no role in the internalisation of soluble B_12_, the data suggests that, to a degree, there might be an element of cross-talk between the two pathways in airway Calu-3 cells. Furthermore, paracellular transport of soluble vitamin B_12_ cannot be ruled out, as suggested for other vitamin B family sub-types [42], which may partly contribute to the observed high level of permeability across the epithelial cells. Similar levels of transepithelial transport have also been reported in intestinal Caco-2 monolayers in studies using comparative (higher than physiological) doses of vitamin B_12_ [43,44].

The cell internalisation route of B_12_-conjugated nanoparticles was probed by the same endocytosis inhibitors used in experiments establishing soluble B_12_ trafficking. The data shows that cell uptake and transport of B_12_-bearing nanoparticles was inhibited by both chlorpromazine and filipin (Fig. 5), implying that these nanoparticles are trafficked by both clathrin-mediated and caveolae-mediated pathways. Interestingly, this finding is notably different to the trends observed for Caco-2 cells, where transport of 50 nm B_12_-conjugated nanoparticles takes place by a route that predominantly involves caveolae [18]. This work therefore not only identifies the presence of biological machinery of vitamin B_12_ trafficking in airway epithelial cells but also demonstrates B_12_-mediated nanoparticle transport into and across the airway epithelium cells — a previously unreported observation. The trafficking of B_12_ bioconjugated nanoparticles in the Calu-3 airway model occurs through different endocytic pathways compared to both soluble B_12_ and unmodified nanoparticles. Demonstration that cell uptake and systemic entry of nano-sized model drug carriers can occur through the airway epithelium *via* the vitamin B_12_ transport system is of potential significant impact in the field of nanomedicine and warrants further research. However, it must be noted that our study was based on a single cell line. The findings will of course have to be confirmed in the future using other airway cell types and, importantly, *in vivo*.

A potential limitation to nanocarrier delivery across the airway mucosa is the presence of mucus. The viscoelastic and adhesive mucus layer that lines the bronchial epithelium presents a barrier to transmucosal movement of nanoparticles and this should be considered when designing nanocarrier delivery systems for systemic delivery through the airways. Particle mobility in mucus is dependent on their surface properties, whereby anionic particles diffuse more efficiently (up to 20–30 times faster) than cationic particles [45] and surface PEGylation remarkably enhances nanoparticle movement in mucus [46,47]. Whilst our work shows the expression, functionality and nanoparticle transport capacity of the vitamin B_12_ pathway in a bronchial epithelial cell line, interestingly, cubilin expression has also recently been demonstrated in human alveolar A549 cells [19]. Assuming that this biological transport pathway is also functional in the alveolar epithelium, it is possible that the deep lung region also contributes to the absorption of B_12_ decorated nanocarriers, which is an interesting prospect considering the enormous surface area of the alveolar epithelium and the lack of mucus barrier in this region. This may be examined in the future *in vitro* using primary human alveolar epithelial cells [48,49], or in animal studies.

## 5 Conclusion

The work investigated the presence of the vitamin B_12_ transport system and its capacity to transport nanoparticles in polarised airway Calu-3 cells. The expression of B_12_ transcytosis component proteins in Calu-3 cells was shown at mRNA and protein level. B_12_-decorated nanoparticles displayed a larger capacity to enter and transport across airway epithelial cell layers (with or without the presence of IF) compared to unmodified nanoparticles. Whilst soluble B_12_ was found to internalise into Calu-3 cells predominantly by a clathrin-mediated route, cell uptake and transport of B_12_-conjugated nanoparticles were dramatically affected by both clathrin and caveolae inhibition, suggesting a different B_12_ intracellular trafficking as a result of its mobilization on nanoparticle surface. The work therefore reveals a previously unexplored route of nanoparticle cell entry and transepithelial transport across the airway epithelium, with a potentially important impact in the area of mucosal delivery of nanomedicines.
