# Supplementary material for: Uptake and transport of B12-conjugated nanoparticles in airway epithelium
Source: J Control Release. 2013 Nov 28;172(1):374–81. doi: 10.1016/j.jconrel.2013.08.028 (PMC3898795; doi:10.1016/j.jconrel.2013.08.028)
Supplement: Supplementary file 1 — Supplementary figures. [file mmc1.docx]

**Supplementary information**

Figure S1. Effect of nanoparticle application on Calu-3 layer transepithelial electrical resistance (TEER). TEER was measured in Hank’s Balanced Salt Solution. Data represents the mean ± SD.

a)

b)

c)

Figure S2. Modification of nanoparticles with B_12_, shown by fluorescence characterization. a) Fluorescence emission (at 546 nm) of 50 nm unmodified nanoparticles with increasing percentage amounts (w/w) of soluble B_12_ b) Fluorescence emission (at 546 nm) showing unmodified nanoparticles and B_12_-conjugated nanoparticles for 50 and 100 nm particle sizes. c) Dialysis experiment showing restored fluorescence intensity of unmodified nanoparticles following removal of free B_12_ and comparison with B_12_-conjugated nanoparticles.

a) b)

**
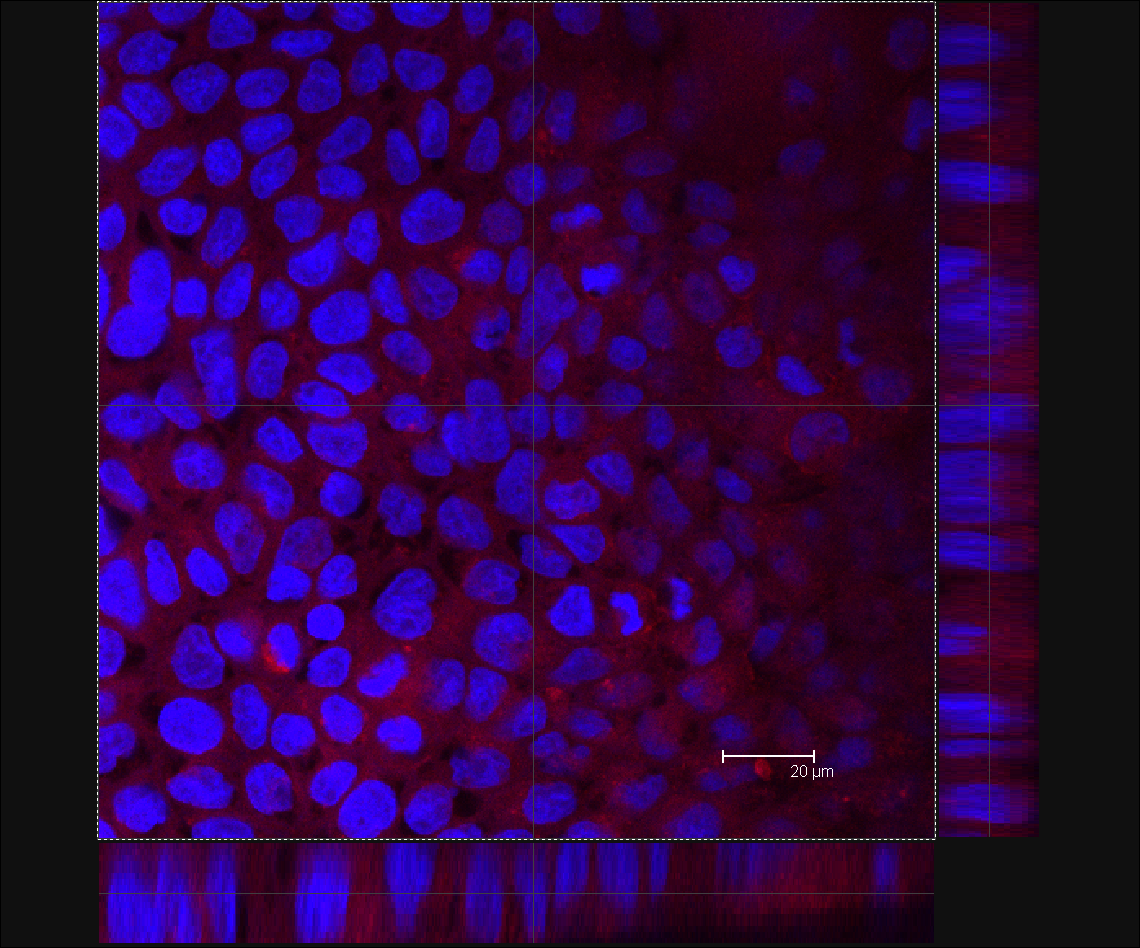

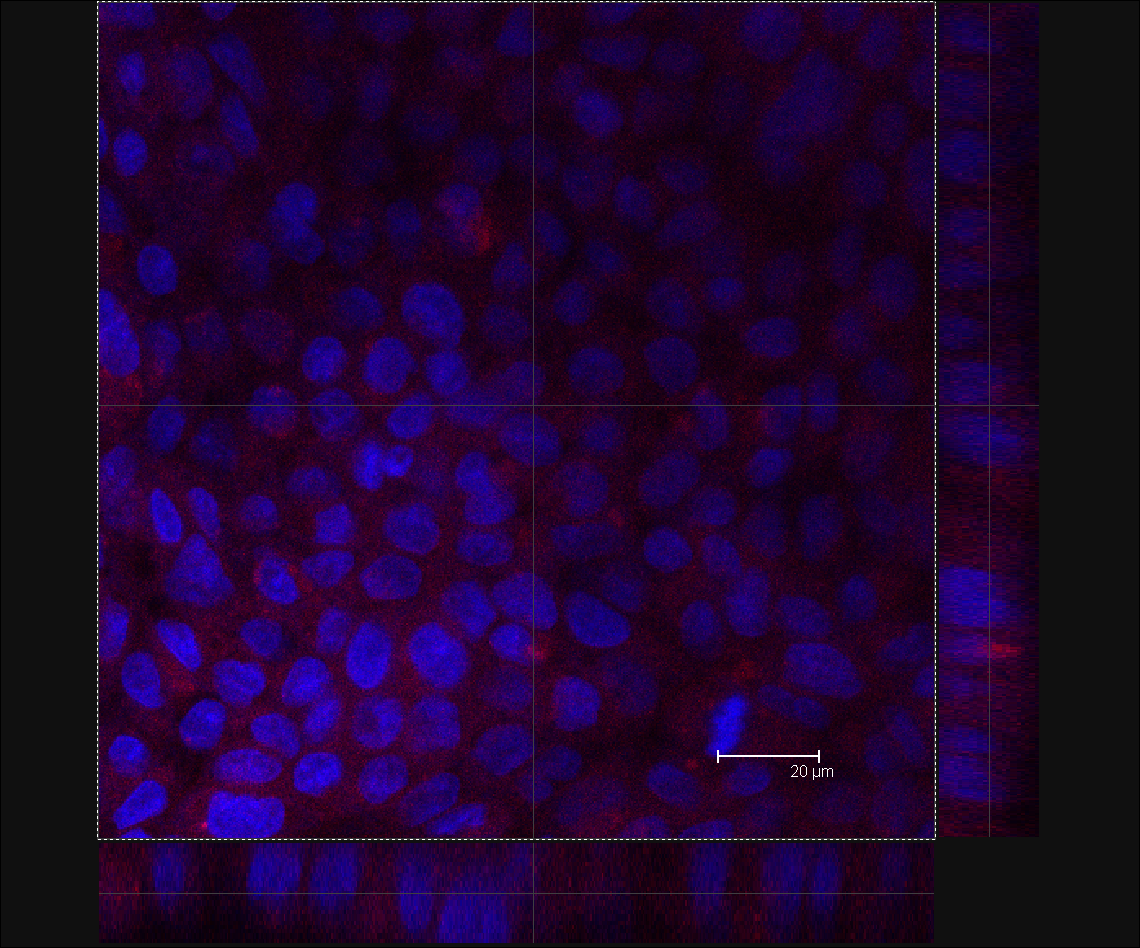
**

Figure S3. Cell uptake of Yellow-Orange nanoparticles. a) unmodified nanoparticles. b) B_12_-conjugated nanoparticles. Cells were labelled for nuclei with Hoechst (blue).

a) b)

**

**

Figure S4. Effect of chemical inhibitors on cell uptake of pathway selective ligands in airway Calu-3 layers. a) Effect of chlorpromazine on uptake of FITC-transferrin, and b) Effect of filipin on cell uptake of Cholera toxin B-subunit.
